# Public attitudes towards cardiopulmonary resuscitation training and performance in Singapore

**DOI:** 10.1186/s12245-021-00378-1

**Published:** 2021-09-15

**Authors:** Susmita Roy Chowdhury, Venkataraman Anantharaman

**Affiliations:** Department of Emergency Medicine, Singapore General Hospital, Outram Road, Singapore, 169608 Singapore

**Keywords:** Attitudes, Bystander, Public, Cardio-pulmonary Resuscitation, Fears

## Abstract

**Background:**

Bystander cardiopulmonary resuscitation (CPR) rates remain fairly low through most communities despite multiple interventions through the years. Understanding the attitudes and fears behind CPR training and performance would help target education and training to raise the rates of bystander CPR and consequently survival rates of victims. 7909 participants at a single-day mass CPR training session in Singapore were given survey questionnaires to fill out. 6473 people submitted completed forms upon the conclusion of the training session. Some issues looked at were the overall level of difficulty of CPR, difficulty levels of specific skills, attitudes towards refresher training, attitudes towards performing CPR, and fears when doing so.

**Results:**

The mean level of difficulty of CPR was rated 3.98 (scale of 1–10), with those with previous CPR training rating it easier. The skills rated most difficult were performing mouth-to-mouth breathing and chest compressions, while the easiest rated was recognizing non-responsiveness. A majority (69.7%) would agree to go for refresher training every 2 years and 88.7% felt everyone should be trained in CPR. 71.6% would perform full CPR for a member of the public in cardiac arrest and only 20.7% would prefer to only do chest compressions. The most cited fear was a low level of confidence, and fears of acquiring infections or aversion to mouth-to-mouth breathing were low.

**Conclusions:**

The survey results show that most participants in Singapore are keen to perform conventional CPR for a member of the public and can help to target future CPR training accordingly.

**Supplementary Information:**

The online version contains supplementary material available at 10.1186/s12245-021-00378-1.

## Introduction

Bystander cardiopulmonary resuscitation (CPR) rates are low in most communities. Over the years, many attempts have been made to increase public involvement in life-saving attempts. In Singapore, beginning 2011, the National Resuscitation Council (NRC) instituted an annual National Life Saving Day (NLSD) on the third Sunday of every year to increase public awareness of life-saving [1]. Since then, bystander CPR rates in Singapore increased from 24.8 to 53.8% [2]. Other interventions also contributed to the increase. Yet, many do not receive bystander assistance when emergencies occur, whether in the home, at work, or in public places. There is a need to understand public attitudes towards CPR training and further improve their commitment to life-saving. The NRC in Singapore was merged with the local National First-Aid Council in April 2018. During the years prior to this merger, the council sanctioned the conduct of a survey of participants at one of the mass CPR training events conducted during an annual NLSD event. The aim of this survey was to determine the attitudes of members of the public attending such training towards the use of this life-saving skill.

## Methods

Participants attending a mass CPR training session conducted by the NRC as part of the NLSD event on a single day at a single venue were issued survey forms to complete at the end of the training. Major training centers in the country assisted in the organization and conduct of the event. Publicity was through the mass media, mailers through community-based organizations, and schools in the vicinity of the event venue. The publicity did not announce the conduct of the survey. At the end of the 3-h CPR training session (which included theory and practical testing followed by certification), the participants were given a survey form which included the following:

- Information on previous CPR training and reasons for learning CPR
- The level of difficulty in learning various CPR skills taught
- Attitude towards refresher training in CPR
- Attitude towards managing family members, members of the public, and work colleagues in managing cardiac arrest
- Fears when performing CPR
- Participants’ suggestions for improving bystander CPR rates in the country

Validation of the survey questionnaire had previously been carried out with ten randomly chosen participants in earlier public CPR training sessions conducted in the three months before the mass CPR event. Feedback from this resulted in amendments to minimize ambiguities and repetitiveness in the questions asked. The revised version was tested with five persons at another small training session just 2 weeks prior to the mass event. No further modifications were needed thereafter. The final version (attached as a supplement) was administered after the mass CPR training event.

Participants were provided pens to complete the survey forms. Participation was voluntary. The participants were not offered monetary or other forms of remuneration for taking part in the survey. Participants were asked to drop their completed survey forms into large marked boxes placed at the exit points of the large event hall where the training event was conducted. The survey forms were then picked up by the event organizers.

The conduct of the survey was approved by the National Resuscitation Council Singapore.

### Data analysis

The data were analyzed with descriptive statistics using SPSS version 21.0 (SPSS Inc., Chicago, IL, USA). Chi-squared testing was used to evaluate differences between discrete groups of participants. A *p* value < 0.05 was used to determine the presence of statistical significance.

## Results

Altogether, 7909 persons participated in the event. Of these 6473 (81.8%) submitted completed survey forms. Males constituted 57.02% of the participants. The mean age of the participants was 20.9 years (Table 1). 67.5% of the attendees were students from a nearby training institution. The remainder were members of the public.

**Table 1:** Age distribution of participants

Age group (years)	Number of participants
9–15	69
16–20	5042
21–30	457
31–40	242
41–50	263
51–60	201
61–80	67
Age not declared	132
**Total**	**6473**

### Previous CPR training

A total of 1730 participants (26.7%) had received previous CPR training. Of these, 49.5% were trained within the last 2 years, 19.9% during the 2 to 4 years prior to the event, and the remainder more than 4 years earlier. 64.4% had wanted to refresh their memory and update their skills to help save a life in an emergency. 12.9% needed the CPR certification as part of their compulsory core-curricular activity in their school or as a job requirement. A further 5.1% needed to renew their CPR certification.

The remainder came because they thought it was either a useful skill to keep, enjoyed attending the training with their friends, or were non-committal on why they attended the training session.

### Level of difficulty

**Fig. 1:**
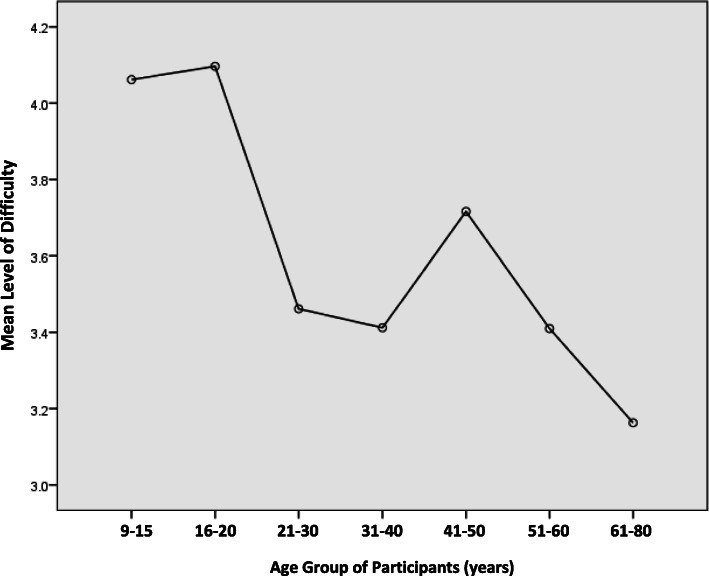
Mean level of difficulty in CPR course. The overall course was rated from a scale of 1 (very easy) to 10 (very difficult). The graph depicts the mean difficulty level rated by each age group

Up to 6111 (94.4%) participants commented on the level of difficulty experienced during the training. On a scale of 0–10, 26.1% found the skills easy (0–2), while 1.7% found them greatly difficult (9–10). The mean level of difficulty was 3.98. Generally, the younger participants had greater difficulty in learning CPR than those in the older age groups (*p*=0.001) (Fig. 1). Those with previous CPR training found the course easier to manage with a score of 3.39 versus 4.21 for those without previous CPR training (*p* <0.001).

The ease of learning five different skills (Table 2) was rated on a scale of 1 (easy) to 5 (very difficult). Recognition of a non-responsive patient was the easiest skill with a score of 1.71 ± 1.17. Next was the ability to recognize the absence of breathing (2.51 ± 1.01) and locating hand position for chest compressions (3.28 ± 1.09). The two most difficult skills were the performance of mouth-to-mouth breathing (MMB) (3.74 ± 1.37) and chest compressions (CC) (3.77 ± 1.19). Participants > 60 years of age had the most difficulty with performing MMB and CC, though they were able to locate the correct hand position for CC with the greatest ease. Those aged 31–40 years had the least difficulty in recognizing lack of responsiveness and absence of breathing.

**Table 2:** Difficulty in learning specific skills during CPR training

Skill	Overall difficulty with specific skills Mean (SD)	Age group (years)								Previous CPR training		
		9–15	16–20	21–30	31–40	41–50	51–60	61–80	***p*** value	Yes	No	***p*** value
**Recognising responsiveness or lack of it**	1.71 (1.17)	1.60 (1.13)	1.74 (1.19)	1.55 (1.07)	1.46 (0.94)	1.52 (1.06)	1.82 (1.28)	1.66 (0.91)	< 0.001	1.67 (1.17)	1.72 (1.17)	0.182
**Recognising absence of breathing**	2.51 (1.01)	2.25 (0.84)	2.53 (1.01)	2.38 (0.94)	2.31 (0.92)	2.43 (0.99)	2.52 (1.08)	2.36 (1.07)	0.001	2.54 (1.00)	2.49 (1.00)	0.071
**Locating hand position for chest compression**	3.28 (1.09)	3.46 (0.94)	3.26 (1.09)	3.41 (1.04)	3.45 (1.04)	3.30 (1.02)	3.05 (1.11)	2.89 (1.17)	<0.001	3.30 (1.07)	3.26 (1.09)	0.220
**Performing chest compressions**	3.77 (1.19)	4.06 (1.10)	3.74 (1.21)	3.83 (1.08)	3.91 (1.08)	3.88 (1.14)	3.96 (1.08)	4.17 (1.04)	0.002	3.75 (1.20)	3.79 (1.19)	0.344
**Performing mouth-to-mouth breathing**	3.74 (1.37)	3.63 (1.32)	3.72 (1.40)	3.84 (1.30)	3.89 (1.19)	3.89 (1.20)	3.68 (1.37)	4.00 (1.06)	0.105	3.74 (1.37)	3.74 (1.37)	0.958

Ease of learning each skill was rated from 1 (not difficult) to 5 (most difficult). This shows the mean ratings stratified by age group and stratified by previous CPR training

### Attitude towards refresher training

Of all participants, 4514 (69.7%) would agree to go for refresher training within the local Singapore framework every 2 years. The commonest reasons for wanting to do so were to refresh and update life-saving skills (57.3%), continue being able to help others and save lives in an emergency (21.7%) and for recertification purposes required by their jobs (5.2%).

For participants who would not want refresher training, 80.6% had no previous CPR training, and 91.6% were <20 years of age. 38.9% felt that having already learnt the skill once was enough. Another 36.9% felt a refresher course would be too troublesome to organize and attend. 19.8% felt they would not have the time to attend a refresher, and a precious Sunday already having been used up away from family and friends.

### Managing family members with cardiac arrest

A total of 5841 participants (90.2%) would perform CC alternating with MMB at the ratio of 30:2 for a family member in cardiac arrest. 4.3% would do only CC and 1.7% only MMB till the arrival of the ambulance. The remainder would not commit themselves as to what they would do. At the same time, 87.2% of the participants would recommend that other members of their family also learn CPR. Of these 56.3% wanted their brothers to learn CPR, 46.7% wanted their sisters, and 60.6% all members of their family to learn the skill.

### Managing members of the public in cardiac arrest

For members of the public, 4637 (71.6%) of participants would perform 30:2 CPR in the event the need arises. 20.7% would do CC only and 1.9% MMB only till the arrival of the ambulance. The remainder would not do any of these. 88.7% of participants wanted all members of the public to be trained in CPR.

### Managing working colleagues in cardiac arrest

Up to 77.5% of the participants would do 30:2 CPR on a working colleague, if in cardiac arrest. 15.5% would have done only CC, and 2.2% only MMB till the arrival of the ambulance. Yet, 10.1% felt their bosses should also be CPR-certified. Only 20.6% wanted their work colleagues to be similarly trained and certified.

### Fears when performing CPR

Nearly 30.0% of the participants expressed some fears when doing CPR (Table 3). Of those expressing fears, 41.5% would, nonetheless, initiate CPR. Of note, only 8.2% expressed fear of acquiring infections during the performance of CPR and 7.6% mentioned aversion to performing MMB.

**Table 3:** Fears when performing CPR

Fears	Frequency (%)
Low level of confidence	960 (41.1%)
Fear of unsuccessful outcome	478 (20.5%)
Fear of causing rib fractures	212 (9.1%)
Fear of acquiring infections	191 (8.2%)
Aversion to doing mouth-to-mouth breathing	178 (7.6%)
Uncomfortable with doing CPR	126 (5.4%)
Legal repercussions of an adverse outcome	58 (2.5%)
Does not want to resuscitate opposite gender	42 (1.8%)
Other fears	90 (3.9%)
**Total**	**2335**

### Suggestions to improve bystander CPR rates

Two thousand two hundred eighty-five of the participants came out with 3898 suggestions to improve community bystander CPR rates (Table 4).

**Table 4:** Suggestions to improve bystander CPR rate in the community

S/No	Suggestion	Frequency
1	Increase number and frequency of public awareness activities	1830
2	Promote CPR for all through various public and private institutions	515
3	Make CPR courses free to the public	486
4	Implement CPR training in all schools	316
5	Have CPR courses easily accessible, such as at community centers	287
6	Have Good Samaritan Laws to help the rescuer	50
7	Have training equipment easily available to the public	48
8	Have AEDs available in more areas in the country	38
9	Provide face-shields to all participants of CPR courses for their keeping	27
10	Raise the profile of successful CPR done by public bystanders	22
11	Others	279

## Discussion

Bystander CPR is a major factor for survival from out-of-hospital-cardiac-arrest (OHCA). This survey was performed to determine the current attitudes and fears, so as to allow targeted education to address the need to improve bystander CPR rates significantly in the near future. To the authors’ knowledge, this was the largest survey done to date in Singapore investigating fears and confidence levels regarding CPR, and willingness to attend further training.

Overall, the skills taught during CPR training were felt to be manageable and not difficult. The difficulty was less amongst those with previous CPR training. This supports the need for refresher courses to maintain CPR skills. A large proportion of participants also indicated an interest in refresher training. This was also echoed in a 2017 UK survey [3] which showed previous training, especially in the previous 5 years, being the most important factor in determining willingness to perform CPR.

Amongst specific skills, recognition of a non-responsive patient was rated to be the easiest. While the use of training manikins might not translate well to a real-world scenario with humans, instructors at this mass event used participant’s training partners as subjects to teach recognition of breathing. This allowed a greater sense of realism for recognition of the factors associated with life, the absence of which would be considered as criteria to begin CPR.

The most difficult skills rated were MMB and CC. This was similarly noted in a Norwegian study [4]. MMB was more difficult in the oldest age group. CC was also more challenging in the extremes of age, likely owing to muscle mass and health-related reasons. CPR training should focus on these practical aspects and maximize the hands-on time to increase confidence in the skills, especially when addressing age groups of concern.

A minority of participants did not see value in repeating the course, already having learnt the skills once. Notably, most of these had no previous CPR training and might not yet be aware of the benefits of refresher training [5, 6]. Repeated and effective CPR training will increase the learner’s willingness and confidence to perform CPR when needed. In addition, CPR training should address the occurrence of skills attrition and the need for refresher training.

A large proportion of participants would recommend their entire family and all members of the public to be CPR-trained. An anomaly however was that only 10% wanted their bosses to be trained in CPR and 20% for their colleagues. A possible reason is the large young non-working group of participants who may not yet appreciate the value of having many trained work colleagues with the skill.

Seventy percent of all OHCA cases occur in residential areas [7] and have lower bystander CPR rates (13.6% vs 38.9%) and poorer survival outcomes—0.9% vs 2.7% rate of survival to discharge, as compared to OHCA in non-residential areas [8]. With the advent of dispatcher-assisted CPR (DACPR), the likely higher rate of bystander CPR in residential OHCA can result in more survivors. Most participants would perform 30:2 CPR for their family members and members of the public. With CPR training more widely carried out, bystander CPR in residential areas can be improved to in excess of 60%. A combination of DACPR, early activation of the emergency ambulance services and self-administered 30:2 CPR will be most likely to lead to improved survival outcomes. Every minute delay in CPR in a patient with cardiac arrest leads to a further reduction in survival [9].

Most participants would perform 30:2 CPR and only 20% would choose to perform CC only. In view of this promising attitude, CPR training for the public should continue to include 30:2 CPR with MMB to optimize conditions for survival. There are multiple conditions in which 30:2 CPR may have better outcomes than CC only, such as drowning, trauma, asphyxia [10], and in pediatric cases [11]. A meta-analysis in 2010 showed dispatcher-assisted CC only to be associated with improved survival compared to dispatcher-assisted 30:2 CPR [12]. This does not mean that non-dispatcher-assisted CC is better than bystander 30:2 CPR. There is still a need to continue standard 30:2 CPR for the training of the public to optimize survival rates and reduce the risk of hypoxia-induced encephalopathy.

The most significant fear expressed was a low level of confidence. This is echoed across many other countries, including Norway [4], Hong Kong [13], Wales [14], and Scotland [15]. Interestingly, the fear of causing injury was only 9%, in contrast to that reported at 22% in Scotland, 28% in Hong Kong, and 22% in Wales. The fear of acquiring infection was only 8.2%, again similar to that reported in Hong Kong at 6.2% and Scotland at 10%. This together with the low reported aversions to do MMB again supports the need to continue with conventional CPR training for the public [16]. We note that the bulk of aversion to MMB in bystander CPR performance comes from health-care workers [17–22].

In terms of legal repercussions, although there is no Good Samaritan law in Singapore, only 2.5% of participants were fearful of this. Conversely in China [23], Hong Kong, and Taiwan [24], the fear of legal action was considerably higher from 14.3 to 53% owing to the perceived lack of a Good Samaritan Law. However, 50 participants suggested to have Good Samaritan Laws available to help the rescuer.

These identified fears can be used to enhance focus on education to reduce these barriers. To combat the low level of confidence, taking into account the higher difficulty reported earlier for the practical skills of MMB and CC, courses can consider increasing time for practical skills. To increase rates of bystander CPR in the community, the suggestions given included public awareness activities, having free and easily accessible CPR courses for the public and implementing CPR training in all schools.

### Limitations

The majority of the participants in this survey were students from a nearby institution, hence the age group of 16–20 was disproportionately represented. While this reduces the generalizability of the survey’s results to the general population, the availability of a relatively large number of older participants is very useful. With the advent of recent mobile-based applications to aid bystander CPR, targeting specific young age groups such as 16–20 might be a good option to increase overall bystander CPR rates. It is also important to understand the fears and attitudes so that appropriate training can help reduce such fears perpetuating into adulthood and becoming learned behaviors.

## Conclusion

This large study supports the need for refresher training and the general willingness of the public to keep their skills refreshed. It also showed participants’ keenness to do conventional CPR even for members of the public. There is a need to continue education during CPR courses to address the fears and concerns raised, primarily a lack of confidence. Starting training at school-going ages might help with long-term attitudes and increase overall bystander CPR rates.

## Supplementary Information

**Additional file 1.** Survey form on attitudes towards Public CPR.

## Data Availability

The datasets generated and analyzed during the current study are not publicly available due to privacy concerns, but are available from the corresponding author at reasonable request.
