# Supplementary material for: Public attitudes towards cardiopulmonary resuscitation training and performance in Singapore
Source: Int J Emerg Med. 2021 Sep 15;14:54. doi: 10.1186/s12245-021-00378-1 (PMC8444401; doi:10.1186/s12245-021-00378-1)
Supplement: Supplementary file 1 — Additional file 1. Survey form on attitudes towards Public CPR. [file 12245_2021_378_MOESM1_ESM.pdf]

SURVEY ON PUBLIC CPR

Personal Particulars:

Name: ………………………………………………………………………… Age: ……………………….

Gender: Male / Female

---

1. Have you undergone CPR training before today? Yes / No. If yes, answer (a) and (b)

   (a) How long ago was it that you last had CPR training. ………………… years

   (b) Why did you want to learn CPR again? ……………………………………………………………

   ……….

   ……….

2. Having completed this CPR course, please state the following:

   a. The level of difficulty in learning CPR was :

   10………9………8………7………6………5………4………3………2………1………0
   (Very difficult) (Very Easy)

   b. The difficult skills to learn were (in order of difficulty)

Most difficult		CPR Skills (Please state number in boxes on left)<br><br>(1) Recognising No Response<br>(2) Recognising No Breathing<br>(3) Locating hand position<br>(4) Performing chest compressions<br>(5) Performing mouth-to-mouth breathing
2nd most difficult		
3rdmost difficult		
4th most difficult		
Not Difficult		

   c. Would you recommend that other members of your family learn CPR?

   Yes / No . If Yes, which members (√) : Brother ◯ Sister◯ Parents◯

d. Would you recommend that other members of the public learn CPR?

Yes / No . If Yes, which members (√) : Colleague ◯ Bosses ◯ All ◯

e. Should CPR training be made compulsory for all levels of working people?

Yes / No . If yes, why? …………………………………………………………………………..
If No, why? …………………………………………………………………………..

3. Would you agree to go for CPR refresher training with recertification in two years?

Yes / No . If yes, why? …………………………………………………………………………..
If No, why? …………………………………………………………………………..

4. If you encounter a family member in cardiac arrest which of the following would you perform?
   (a) only chest compressions till the ambulance arrives
   (b) only mouth-to-mouth ventilations till the ambulance arrives
   (c) both chest compressions and mouth-to-mouth ventilations at 30 : 2 till ambulance arrives
   (d) none of the above

5. If you encounter a member of the public in cardiac arrest which of the following would you perform?
   (a) only chest compressions till the ambulance arrives
   (b) only mouth-to-mouth ventilations till the ambulance arrives
   (c) both chest compressions and mouth-to-mouth ventilations at 30 : 2 till ambulance arrives
   (d) none of the above

6. If you encounter a work colleague in cardiac arrest which of the following would you perform?
   (a) only chest compressions till the ambulance arrives
   (b) only mouth-to-mouth ventilations till the ambulance arrives
   (c) both chest compressions and mouth-to-mouth ventilations at 30 : 2 till ambulance arrives
   (d) none of the above

7. Do you have any fears when doing CPR? Yes / No

8. If yes, list some of these fears:

   (a) …………………………………………………… (b) ……………………………………………………

   (c) …………………………………………………… (d) ……………………………………………………

9. Would you not do CPR or delay CPR because of these fears? Yes / No

10. What suggestions do you have to improve rate of bystander CPR in the community?

    a. ……..
    b. ……..
    c. ……..
    d. ……..
    e. ……..
